# Misinformation, believability, and vaccine acceptance over 40 countries: Takeaways from the initial phase of the COVID-19 infodemic

**DOI:** 10.1371/journal.pone.0263381

**Published:** 2022-02-09

**Authors:** Karandeep Singh, Gabriel Lima, Meeyoung Cha, Chiyoung Cha, Juhi Kulshrestha, Yong-Yeol Ahn, Onur Varol

**Affiliations:** 1 Institute for Basic Science, Daejeon, South Korea; 2 School of Computing, KAIST, Daejeon, South Korea; 3 College of Nursing, Graduate Program in System Health Science and Engineering, Ewha Womans University, Seoul, South Korea; 4 Department of Politics and Public Administration, University of Konstanz, Konstanz, Germany; 5 Center for Complex Networks and Systems Research, Luddy School of Informatics, Computing, and Engineering, Indiana University, Bloomington, IN, United States of America; 6 Indiana University Network Science Institute, Indiana University, Bloomington, IN, United States of America; 7 Connection Science, Massachusetts Institute of Technology, Cambridge, MA, United States of America; 8 Faculty of Engineering and Natural Sciences, Sabanci University, Istanbul, Turkey; National Research Council (CNR), ITALY

## Abstract

The COVID-19 pandemic has been damaging to the lives of people all around the world. Accompanied by the pandemic is an *infodemic*, an abundant and uncontrolled spread of potentially harmful misinformation. The infodemic may severely change the pandemic’s course by interfering with public health interventions such as wearing masks, social distancing, and vaccination. In particular, the impact of the infodemic on vaccination is critical because it holds the key to reverting to pre-pandemic normalcy. This paper presents findings from a global survey on the extent of worldwide exposure to the COVID-19 infodemic, assesses different populations’ susceptibility to false claims, and analyzes its association with vaccine acceptance. Based on responses gathered from over 18,400 individuals from 40 countries, we find a strong association between perceived believability of COVID-19 misinformation and vaccination hesitancy. Our study shows that only half of the online users exposed to rumors might have seen corresponding fact-checked information. Moreover, depending on the country, between 6% and 37% of individuals considered these rumors believable. A key finding of this research is that poorer regions were more susceptible to encountering and believing COVID-19 misinformation; countries with lower gross domestic product (GDP) per capita showed a substantially higher prevalence of misinformation. We discuss implications of our findings to public campaigns that proactively spread accurate information to countries that are more susceptible to the infodemic. We also defend that fact-checking platforms should prioritize claims that not only have wide exposure but are also perceived to be believable. Our findings give insights into how to successfully handle risk communication during the initial phase of a future pandemic.

## Introduction

In the contemporary world with social media, misinformation and disinformation can be rapidly disseminated to millions of people [1, 2]. Studies suggest that false information spreads more broadly than the truth online [3]. Due to social media’s global reach with a rapid amplification mechanism [4], information can quickly inundate the Internet and get reinforced, potentially creating an “infodemic” [5, 6]. This abundance of information can lead to harmful consequences. For instance, the COVID-19 infodemic has resulted in seemingly harmless acts such as shaving one’s head and saltwater gargling [7], but it has also led to illegal and damaging acts like arson [8].

Previous work addressing misinformation in healthcare has found that false and misleading claims negatively influence people’s attitudes towards vaccines. One study conducted in the Democratic Republic of the Congo during the Ebola epidemic found an adverse effect of false information on vaccine acceptance [9]. The World Health Organization (WHO) has highlighted how misinformation has raised doubts on the effectiveness of human papillomavirus (HPV) vaccines [10]. Vaccine refusal has led to the measles’ resurgence in the US, even after decades of containment [11]. In the context of the coronavirus pandemic, a vaccine is widely believed to be the only way out towards pre-pandemic normalcy [12].

Due to the Internet’s nature, it is challenging to prevent the spread of false information [13]. There is no established authority that checks the veracity of the information that is shared online. Moreover, social media can quickly spread a piece of information to large groups of people, independently of its source and authenticity. Misinformation, disinformation, and eccentric opinions can get reinforced by repeated exposure and thus threaten public health. As a result, communicating even the most basic facts to the public can become a challenge.

A possible remedy to the harm caused by an infodemic is flagging and removing false information online. Extensive research has focused on automating this process [14, 15], and social media platforms have recently taken both proactive and reactive steps to prevent and minimize the spread of misinformation [16, 17].

Proactive dissemination of fact-checked information preempting the spread of the infodemic is another way to combat misinformation. Working on these lines, we launched an online campaign to debunk COVID-19 rumors [18] that disseminated accurate coronavirus-related information to over 50,000 individuals. The campaign aimed to collect fact-checked information from regions that had already suffered from the infodemic and spread them to other regions where the infodemic was at its infancy.

**Fig 1:**
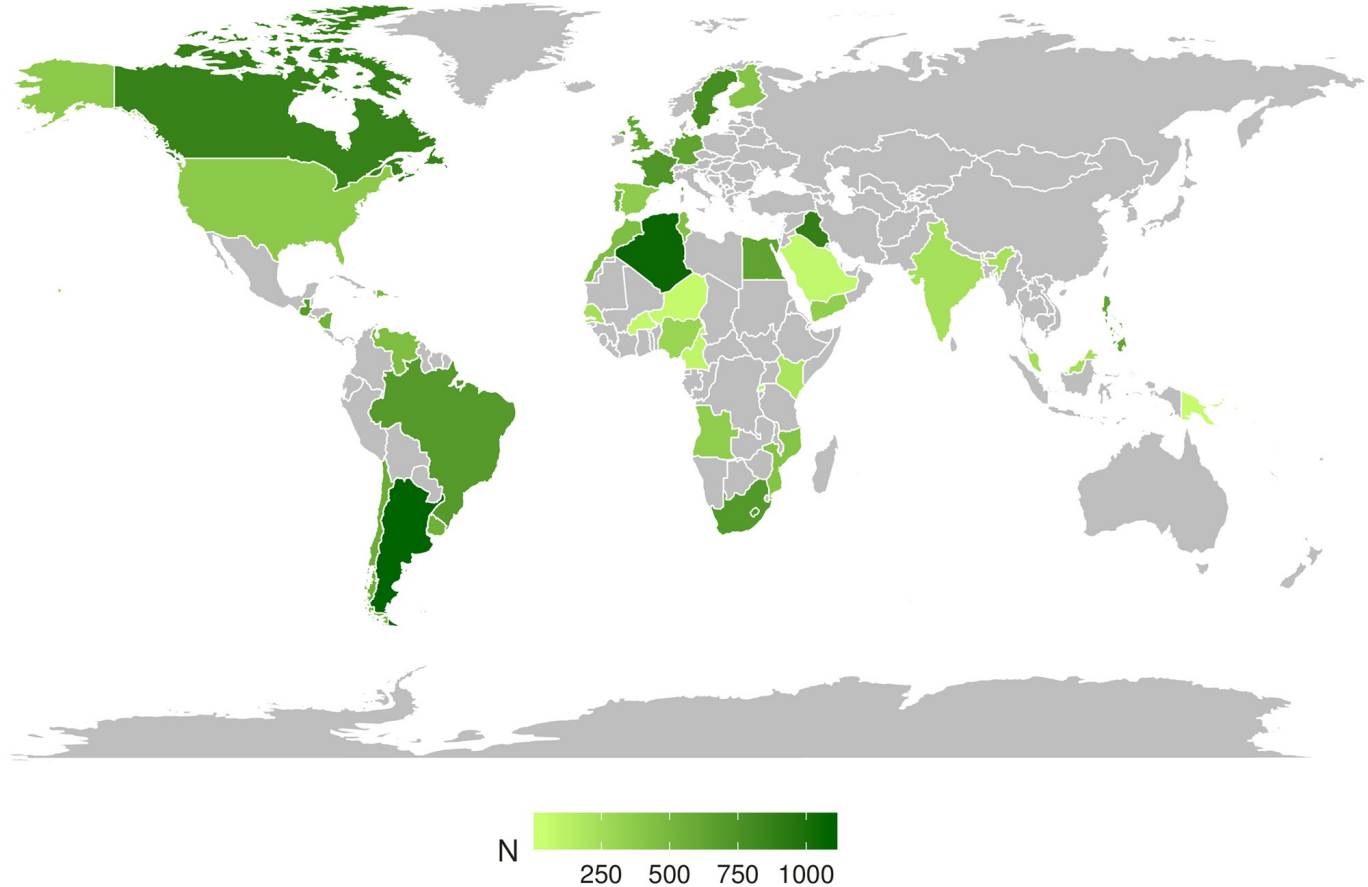
Distribution of study participants around the world. The study obtained responses from 18,407 participants from 40 countries.

Many studies have addressed the pressing issue of the COVID-19 infodemic [19–22]. These studies targeted the problem either from a computational or exploratory perspective. In this work, we employed a survey-based approach to analyze the prevalence of the COVID-19 infodemic. We use the data gathered from our public campaign and study the public’s susceptibility to the infodemic. Through a global-scale survey conducted using the Facebook Advertisement Platform, we collected 18,407 complete responses from individuals in 40 countries (see Fig 1) and measured the extent to which a wide range of coronavirus-related rumors and their respective fact-checks reached different countries. We also examined the association between exposure to misinformation, perceived believability of false claims, and vaccine hesitancy at an individual level.

We find that distinct claims disseminated differently around the world, disproportionately affecting less economically developed countries. Our results also indicate that false information is nearly two times more prevalent than corresponding fact-checked information, indicating that half of the online users exposed to rumors might not have encountered the corresponding fact-checks.

When jointly considering rumors’ perceived believability, the picture becomes more complicated. Although some claims—such as those 5G-related—exhibit inherently low believability, we find that they spread widely. On the other hand, other popular claims, e.g., regarding the use of existing drugs, have relatively high believability. This finding implies that fact-checking organizations could utilize user response to quick polls to identify claims that are more likely to be widely believed. Such a prioritization strategy could be helpful given fact-checking systems’ limited resources.

Our study found a positive association between exposure and both believability and vaccination hesitancy. The results also show that those who perceive the pandemic as more threatening are more willing to get vaccinated, highlighting the importance of raising public awareness concerning the disease’s risks. Our regression analysis suggests that exposure to fact-checks could nearly balance out the adverse effect of exposure to misinformation. This remedy, however, does not seem to be effective for individuals that are susceptible to false information; to what extent participants found claims believable was much more strongly associated with vaccine hesitancy.

Given the rising social media usage, including in developing and underdeveloped regions, social media platforms could be used as a primary medium for disseminating fact-checks. We propose one algorithmic prioritization method for future debunking strategies that account for the varying degrees of claim believability and spread. This work demonstrates how web data can be analyzed to understand important health implications during a global pandemic. We describe our methodology for conducting surveys over a social media platform and post-processing the data to correct for sampling demographic biases.

## Materials and methods

### COVID-19 claims selection

For identifying popular false claims, we collected over 200 COVID-19 rumors from DXY.cn, a Chinese online community for physicians and healthcare professionals, on March 18, 2020. This site hosted a comprehensive list of Chinese social media rumors during the COVID-19 infancy in China. Many of them were later found to have spread worldwide. After removing redundant content and lockdown-related claims, we investigated 30 pieces of misinformation addressing health-related behaviors. We combined these pieces into 11 distinct claims and categorized claims into subgroups depending on the rumor’s nature (e.g., those addressing vaccination or do it yourself (DIY) measures). Corroborating these claims with fact-checked information from credible sources, including the World Health Organization (WHO)’s Mythbusters [23] and the International Fact-Checking Network (IFCN)’s #CoronaVirusFacts Alliance database [24], we arrived at the following list of 11 false claims:

1. 5G (5G): 5G networks can contribute the spreading of the coronavirus.
2. Dryer (Hot&Co): Hot-air dryers can kill the coronavirus.
3. Gargling (DIY): Gargling with salt water can prevent coronavirus infection.
4. Drugs (DIY): Existing drugs for malaria and HIV can help treat COVID-19.
5. Pharma (Vaccination): Pharmaceutical companies are spreading COVID-19 so they can profit from its vaccine.
6. Population (Vaccination): The COVID-19 vaccines currently being developed are forms of population control.
7. Sunbath (Hot&Co): Standing in the sun can kill the coronavirus.
8. Tracking (Vaccination): The COVID-19 vaccine is being developed to implant people with tracking microchips.
9. Vinegar(DIY): Apple cider vinegar can kill the coronavirus in the throat.
10. Water (DIY): Drinking water every 15 minutes will prevent getting infected with the coronavirus.
11. Weather (Hot&Co): The coronavirus will only spread in cold, dry weather and does not survive in hot, humid weather.

Our survey and claims were translated into English, French, Spanish, Portuguese, and Arabic. For French and Spanish, we first used Google Translate to obtain crude translations and then used the Prolific crowdsourcing platform [25] to recruit native speakers from these languages (minimum 18 each) to refine the translations. Recruited participants attended a short survey in which they were asked to refine the provided translations. This procedure was repeated three times for each language in an iterative manner. The translations were done entirely by volunteering native speakers from the co-first author’s institution for Portuguese and Arabic.

### Survey design

This study was approved by the Institutional Review Board at KAIST (IRB-20–229) and performed in accordance with the relevant guidelines and regulations. Informed consent was obtained from all study participants. We only recruited participants older than 18 years of age. Participants were presented with the following text before taking part in the study:

Thank you for your interest in this survey. This survey will present you with some information regarding COVID-19 that has been shared on social media. We would like to know what you think about them. We are not looking for the correct answers, but your opinion on the matter. Please, read all the information provided in this survey carefully.

The purpose of this research is to understand how people perceive information regarding COVID-19. This experiment is composed of an online survey that takes about 10 minutes to complete. The information we collect will be used only for research purposes and will be kept in secure computer files indefinitely. No names or other identifying information will be used in any publications or presentations that may result from this study. Your responses may be shared with other researchers; all information, however, will be anonymized and allow no inference on any particular individual.

Results will be published only as aggregate statistics, allowing no inference on any particular individual. Your participation is voluntary and you may withdraw from the study at any time without any penalty. Your responses will be analyzed regardless of how many questions you have completed.

Clicking next indicates that you have understood the information and consent to your participation.

Our survey was designed to address four different aspects of the coronavirus infodemic to the public: *i*) exposure to misinformation, *ii*) exposure to fact-checks, *iii*) perception of claim believability, and *iv*) perception of how beneficial fact-checks could be to one’s community. The following questions were presented in random order to the survey participants for each of the claims:

1. *Exposure*: Have you seen or heard this information in the last month? (Answer: Yes, Partly, No, I don’t remember)
2. *Fact-Checks*: Have you ever seen an official source confirming or denying the claim above? (Answer: Yes, No, I don’t remember)
3. *Believability*: How believable does the information above seem to you? (Answer: Not believable at all, Not really believable, I am not sure, Somewhat believable, Very believable)
4. *Benefit*: To what extent would your community benefit from seeing a fact-checking result of the claim above? (Answer: Not at all, A little, Moderately, A lot)

Participants were not explicitly asked whether they believed the study’s rumors to avoid potential social desirability biases. Respondents might have answered that they did not believe the claims so that they would be seen more favorably. Hence, we phrased our questions such that they were asked to what extent they found the rumors to be believable. Note that our study measures people’s susceptibility to believing in misinformation, i.e., their perception of the claims’ believability, not their actual belief. At the end of the survey, respondents were also asked demographic questions and to what extent they perceived the novel coronavirus as a threat, measured via a threat scale introduced in [26] (termed *perceived threat*). All questions were translated into English, French, Spanish, Portuguese, and Arabic using the same procedure employed for claims.

### Data collection

We conducted a large-scale online survey using the Facebook Advertising Platform from June 18 to July 13, 2020. The survey was designed in the SurveyMonkey platform, and the link to the survey was made available via advertisements on Facebook. As of March 2020, Facebook had 2.60 billion monthly active users and 1.73 billion daily active users [27], making it the largest social media platform. Some recent publications [28, 29] have explored Facebook’s usage as a survey platform and noted its deep and broad reach, rapid data collection, granular targeting, and cost-effectiveness. The Facebook Advertising Platform allows targeting based on age, location, spoken language, and a range of user interests. Sample biases can be dealt with the adequate application of post-stratification weighting techniques, although studies such as [30] show that the demographic distributions of Facebook users tend to not differ hugely from national censuses.

To obtain a larger and more representative sample of every country, we designed independent Facebook campaigns for the target countries. Each campaign was further divided into four advertisement sets by age groups (18–24, 25–44, 45–64, and 65+ years). The English survey was available from June 18 to June 25, the Portuguese, Spanish and French surveys were conducted from June 22 to June 28, while the Arabic survey was run from July 7 to July 13.

To control for demographic factors, we ensure a minimum sample of 100 responses from each country. For countries with less than 30 complete responses from the initial round, the survey was rerun from July 7 to July 13. Since the respondents were recruited through the Facebook Advertising Platform, they did not receive any financial benefit from participating in the survey. Participation was voluntary, and respondents could choose to withdraw from the survey at any time.

We obtained 1,946,516 responses (*N* = 44,239) from Facebook users who saw and clicked on our advertisement. We discarded incomplete responses and participants with duplicated IP addresses. Due to our weighted analysis, which requires each participant to report their sex, age, and country of residence, we discarded responses from participants who chose not to reveal their sex. We allowed participants to report their sex as “other.” To weigh responses successfully, we solely kept participants from those countries with at least 30 complete responses. Our final dataset consisted of 805,816 complete responses (*N* = 18,314) from 40 countries (see Fig 1). Our sample covers all continents and contains a median of 464 respondents per country. Demographic information regarding all participants is presented in the S1 Table in S1 File.

### Sample and weighting

Recruiting participants through the Facebook Advertising Platform allowed us to reach a larger and more representative pool of respondents than otherwise possible through crowdsourcing platforms. Nevertheless, Facebook users are still not demographically representative of countries’ populations. For instance, although previous work has found a high correlation between the US Census and Facebook users, the latter was composed of younger and more educated people [30].

To compensate for any imbalance between our sample and the general population demographic distributions, we employed *raking* as a post-stratified weighting technique. Raking is an iterative method that calculates weights for each joint demographic group until convergence [31]. These weights can be used to estimate a population’s information more accurately given a non-representative sample. Previous research has shown that Facebook user demographics are comparable to gold-standard surveys and the differences can be dealt with by appropriate weighing techniques [30].

After obtaining estimates of each country’s age and sex distributions from the United Nation’s 2019 World Population Prospects dataset, we used raking for calculating each response’s weight. This technique was employed for each country, resulting in weights for each sex, age group, and country triple in our dataset. As the UN provides information about age distributions in 5-year intervals, we used the 20–24 bracket corresponding to our 18–24 age group for weighting purposes. Unless stated otherwise, we present weighted results from our analysis.

**Fig 2:**
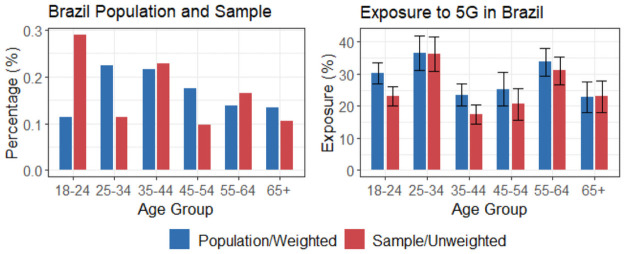
Sample and population age distributions for Brazil (*N* = 698). As an example, this figures highlights the differences between weighted and unweighted exposure rates to the 5G claim. Error bars are standard errors.

Fig 2 exemplifies the weighing process. The left plot shows our sample’s age distribution from Brazilian survey respondents alongside the true Brazilian population’s age distribution. It is possible to note that our survey sample is younger than the real population. Weighting techniques like raking compensate for this discrepancy and help obtain a more accurate population-level estimation [31]. The right plot in Fig 2 shows how weighting adjusted the sample’s estimates for the case of exposure to the 5G rumor. Some age groups exhibit statistically significant differences between the weighted and unweighted estimates.

### Regression models

This work presents three different regression models, all of which consider reported demographics features and the respondents’ mean perceived threat as control variables. The demographic features in this study are reported age group, sex, education, health, and financial status. Except for sex and vaccination history-related dummies, all variables are treated as continuous or counts (see S2 Table in S1 File). Our independent variables correspond to exposure to misinformation and the respective fact-check counts for Model 1, and additionally, average believability for models 2 and 3. We add interaction terms for vaccination history (as history for a non-mandatory vaccine implies past vaccination) as well for exposure to claims and their respective fact-checks (to be exposed to a fact-checked also implies that one has been exposed to the claim, even if only at the time of debunking).

Model 1 is a linear regression predicting the average reported believability of false claims. It is of the form:

$$\begin{array}{c} Y∼\alpha +\sum_{i}\beta_{i}.C_{i}+\sum_{g}\beta_{g}.I_{g}+\sum_{k}\beta_{k}.T_{k} \end{array}$$ (1)

where *Y* is the mean believability, *α* is the intercept term, and *β*_*i*_, *β*_*g*_, *β*_*k*_ are coefficients for control variables *C*_*i*_, independent variables *I*_*g*_, and interaction terms *T*_*k*_ respectively.

Model 2 and 3 are logistic regression models predicting the dichotomized responses to the COVID-19 vaccine acceptance question (with the third option “I don’t know” treated as a negative response). In order to segregate and identify the association of different categories of claims with vaccination acceptance, we divided the claims into four groups in Model 3: DIY, Hot&Co, vaccination conspiracies, and 5G-related claim. For Model 3, the independent variables are distinct exposure and fact-check counts and the mean believability of the segregated groups, whereas we aggregate these variables across all claims for Model 2.

Model 2 and 3 are of the form:

$$\begin{array}{c} \text{log}\frac{P}{1-P}∼\alpha +\sum_{i}\beta_{i}.C_{i}+\sum_{c,g}\beta_{cg}.I_{cg}+\sum_{h}\beta_{h}.V_{h}+\sum_{k}\beta_{k}.T_{k} \end{array}$$ (2)

where *P* is vaccine acceptance, *α* is the intercept term, *β*_*i*_, *β*_*cg*_, *β*_*h*_, *β*_*k*_ are coefficients for control variables *C*_*i*_, independent variables *I*_*cg*_ (representing question categories *c* and variable *g*), vaccination history *V*_*h*_, and interactions terms *T*_*k*_ (as explained above), respectively. We present results with country-level random effects and with lasso and elastic regularization in the S3–S14 Tables in S1 File.

## Results

### Exposure to rumors

**Fig 3:**
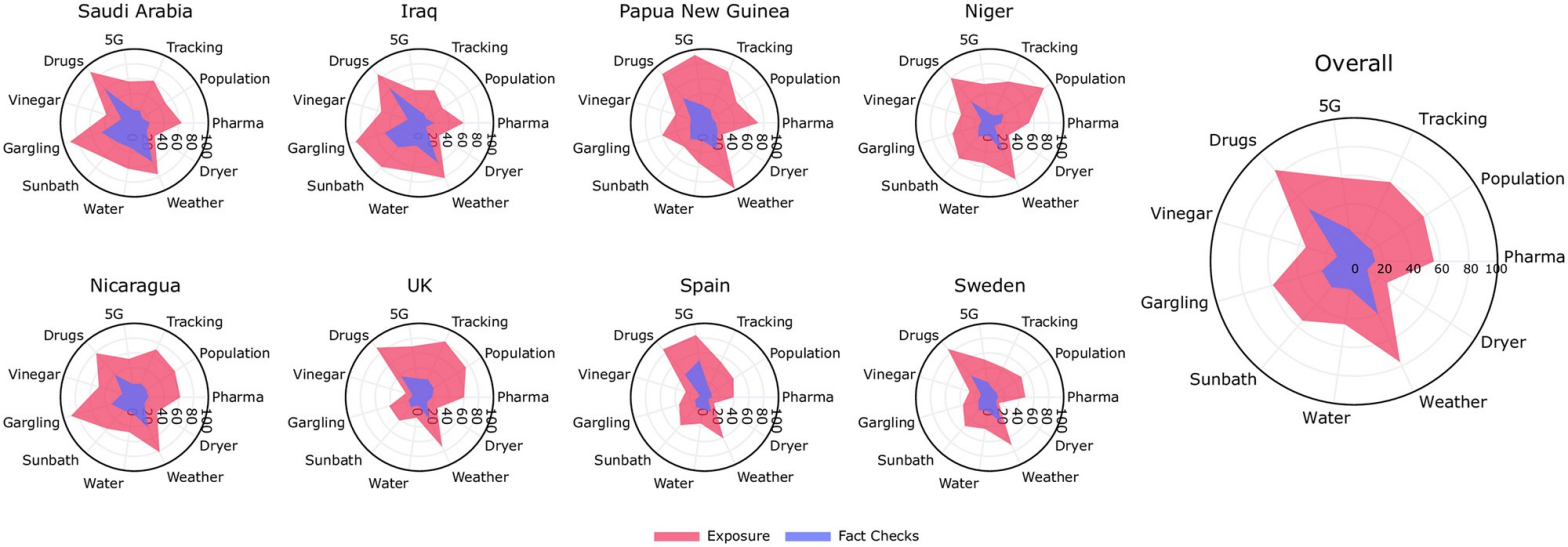
Country-level exposure to rumors and fact-checks. The pink polygon presents the weighted percentage of people who have been exposed to rumors. The purple polygon shows exposure to fact-checks.

Fig 3 shows to what extent different countries have been exposed to COVID-19 misinformation and their respective fact-checks. The countries presented in Fig 3 were selected to cover different regions of the world and varying exposure levels to the claims. We also show the overall weighted average across all 40 countries. All others countries are presented in the S1 Fig in S1 File.

When we examine the overall exposure, we note that some claims have exceptionally high public appeal.Drugs and Weather are the two most popular claims, with an average of 84.2% and 77.1% of participants encountering these rumors, respectively. Vaccination-related claims show moderate-to-high exposure with Tracking being seen by 60.7%, Population by 57.6% and Pharma by 55.7% of the respondents. A smaller portion of respondents were exposed to do-it-yourself (DIY) rumors on preventive measures with Sunbath seen by 54.7%, Water by 44.3%, Vinegar by 34.9%, and Dryer by 27.4% of the respondents.

Next, we investigated the COVID-19 infodemic’s reach across different geographical regions by comparing the pink polygons in Fig 3 representing the extent to which a country has been exposed to the infodemic rumors. Countries in the same region are exposed to the claims to similar extent (exemplified by a similar shape of the polygon for the Middle Eastern countries of Saudi Arabia and Iraq, or the European nations of the UK, Spain, and Sweden). At the same time, there is a noticeable variance in the infodemic’s reach across continents.

The selected claims also demonstrate varying levels of exposure in different countries. Some rumors are regionally concentrated. For instance, Gargling was widely disseminated in Saudi Arabia, Iraq, and Nicaragua (i.e., above 90%), with markedly low exposure in the UK, Spain and Sweden (i.e., 40% or less). Similarly, 5G was seen widely in Papua New Guinea, Spain, and the UK, but less in other regions. On the other hand, rumors regarding Drugs, Weather, and vaccine-related claims (Tracking, Population, and Pharma) spread globally and received significant exposure across countries.

### Exposure to fact-checks

The inner purple polygons in Fig 3 show the extent to which participants were exposed to fact-checks for each claim covered by our study. Our results reveal that 48.6% of participants were exposed to fact-checks on the Drugs rumor, which had spread the highest. The second most popular claim, Weather, was also the second most fact-checked claim, with 40.3% of respondents seeing an official source confirming or denying it. Fact-checks for all other claims had been seen by no more than 25% of respondents on average.

Our results demonstrate that fact-checks do not spread at the same rate as the rumors themselves. On average, fewer than half the respondents who had seen a rumors had encountered its corresponding fact-check, as demonstrated by the difference in areas of pink and purple regions in Fig 3. Given that we opted to choose prevalent rumors on social media, we highlight that only less than half of the people seeing the corresponding fact-checked information is quite alarming. Finally, we investigated the relationship between the perceived benefit of sharing fact-checks and participants’ exposure to them. Our results suggest that respondents perceive fact-checks to be more beneficial to their community if they address less commonly seen rumors (Spearman’s *ρ* = -0.745, 95% CI -0.972 − -0.120).

### The most vulnerable regions

**Fig 4:**
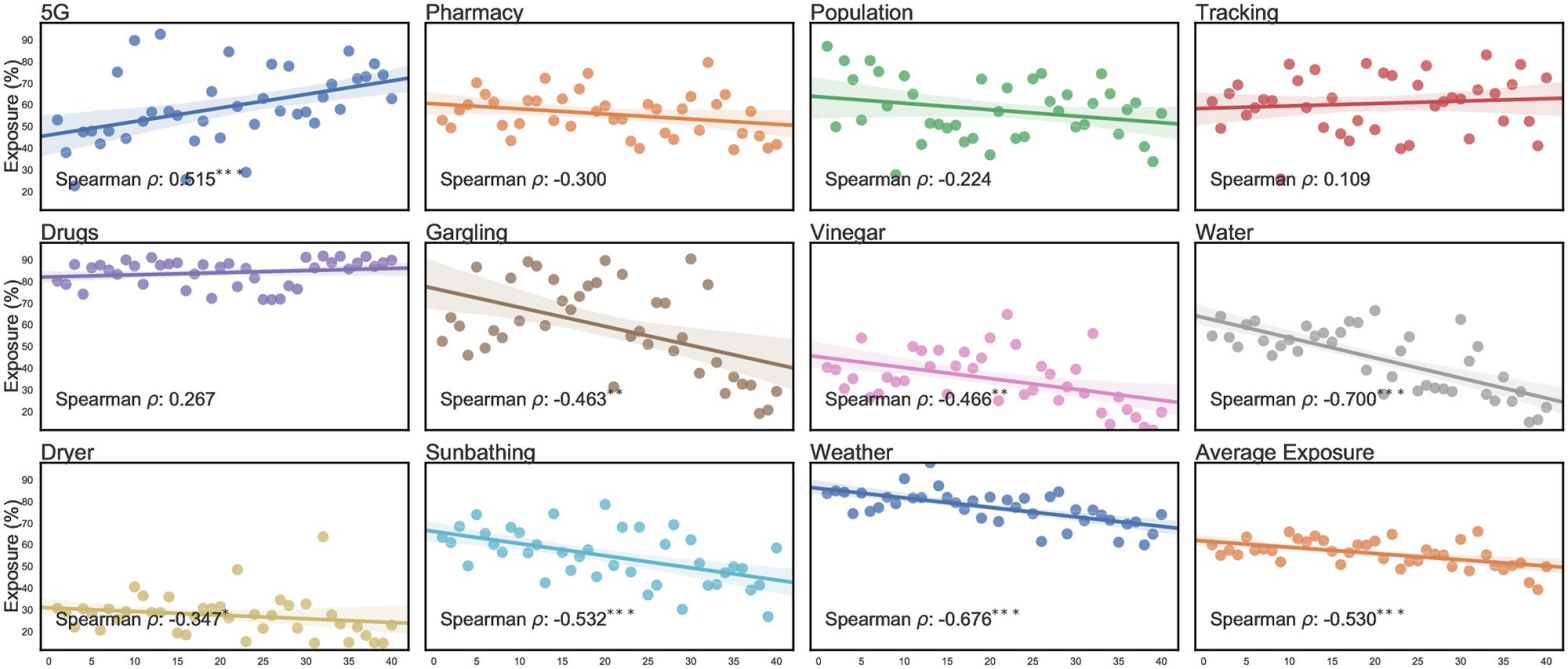
Scatter plot and linear relationship between country-level exposure to rumors and ranked GDP per capita. The x-axis represents the ranked GDP per capita values of countries in our study. Spearman correlation values and their respective significance levels are also presented. The rightmost bottom plot presents the results across all claims. Significance marked as * *p*<.05, ** *p*<.01, *** *p*<.001.

Fig 4 indicates a relationship between the infodemic’s reach and a country’s economic development; developed countries (e.g., Sweden, Spain) appear to have been less exposed to the infodemic than underdeveloped or developing countries (e.g., Iraq, Nicaragua). Fig 4 shows the level of exposure to each of the eleven rumors by country while ordering countries by their GDP per capita on the x-axis. The choice of GDP per capita is motivated by the fact that it is the most widely used comparative economic indicator. We also assume it as a proxy for health indicators and healthcare infrastructure.

Our results indicate that the 5G claim was seen more widely in developed countries than in other regions (*ρ* = 0.515, 95% CI 0.248 − 0.779). On the other hand, vaccination-related—Pharmacy, Population, and Tracking—and the Drugs claims show no significant difference in exposure between developed and underdeveloped countries. For the remaining claims, we observe a downward trend (i.e., negative correlation), suggesting that disadvantaged nations are more vulnerable to the infodemic. These claims include Weather (*ρ* = -0.676, 95% CI -0.863 − -0.485) and DIY measures such as Vinegar (*ρ* = -0.466, 95% CI -0.777 − -0.153), Sunbath (*ρ* = -0.532, 95% CI -0.753 − -0.309), Gargling (*ρ* = -0.463, 95% CI -0.786 − -0.136), Water (*ρ* = -0.700, 95% CI -0.905 − -0.491), and Dryer (*ρ* = -0.347, 95% CI -0.658 − -0.035).

### Believability of rumors

As a measure of people’s perception of these claims, we asked survey respondents to indicate the extent to which they found each claim to be “believable” on a 5-point Likert scale. The responses ranged from ‘Not believable at all’ (score of -2) to ‘Very believable’ (score of 2). The only claim perceived to be somewhat believable was the Drugs rumor, with a weighted mean value of 0.190 (i.e., borderline believable). All other claims reported a low mean believability (below -0.5), with the 5G claim being the least believable claim with a mean value of -1.307.

To understand what fraction of respondents from each country might be susceptible to believing in misinformation, we dichotomized the reported believability values into positive (i.e., susceptible to believing in a rumor) and negative (i.e., not susceptible). On average, we observe that 22% of respondents per country are predisposed to believing in rumors. We note the highest misinformation believability (more than 31%) in Yemen, Algeria, Saudi Arabia, and Tunisia. Swedish and Finnish people seem the least susceptible to the infodemic, with a mere 7.4% of respondents reporting that rumors are believable.

**Fig 5:**
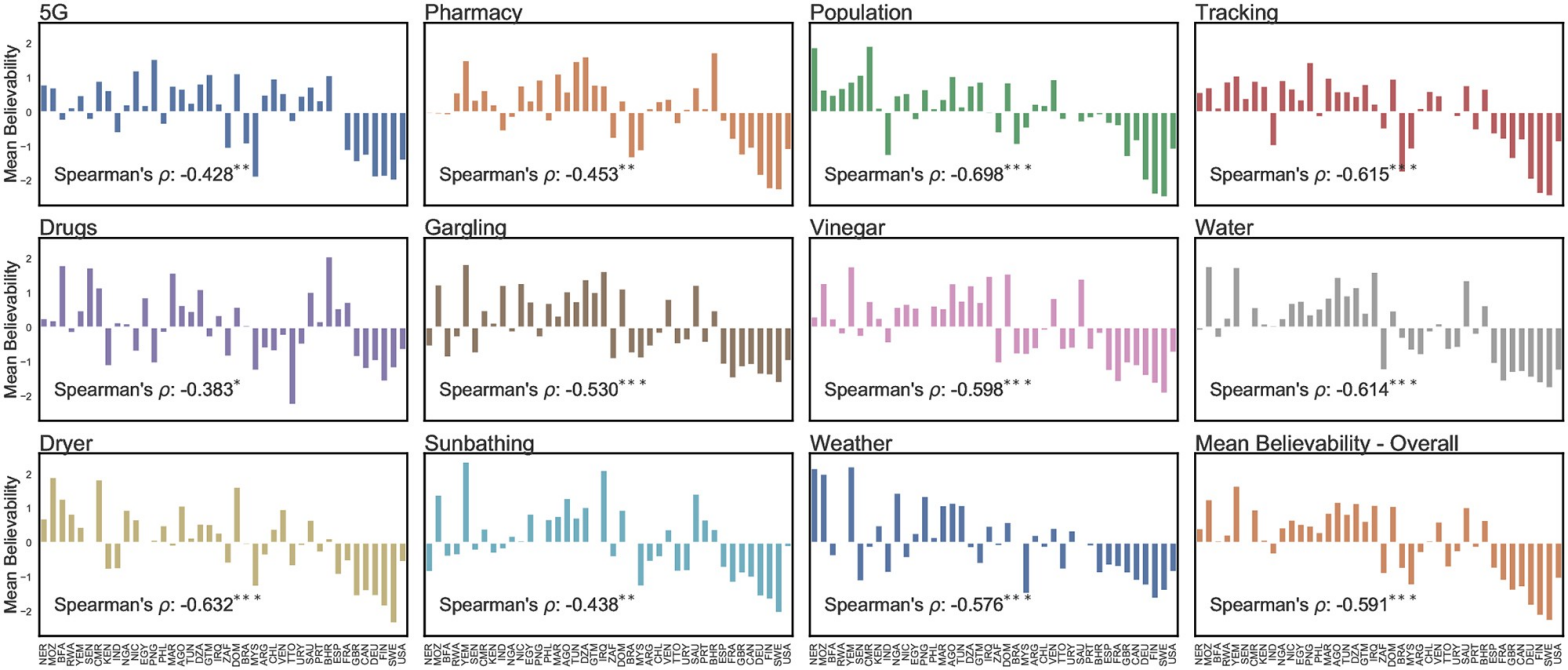
Relative perceived believability of each rumor addressed in this study. Country-level z-scores are presented. The countries on the x-axis are in increasing order of GDP per capita.

After determining a country’s weighted average for each claim, we calculated a nation’s believability z-score. Fig 5 presents every country’s perceived believability (with the countries ranked in increasing order of their GDP per capita) for each rumor. A positive value suggests that a country’s population finds a specific claim more believable than an average community, i.e., is more susceptible to believing in it.

A visual inspection of Fig 5 reveals that the most economically developed countries covered by our study show the lowest rates of believability across all claims. Conversely, the lower half of the nations ranked by GDP per capita appear to have the highest believability values. These findings suggest that misinformation is likely to be perceived as more believable in economically vulnerable countries.

### Misinformation and vaccine acceptance

After quantifying the public exposure to misinformation, we assessed the perceived believability of each rumor (termed *believability* hereafter) and its relationship to willingness to get vaccinated (termed *vaccine acceptance*). Our primary goal was to examine whether extensive exposure to misinformation increases rumor believability and whether such reinforcement further leads to vaccine hesitancy (i.e., a decrease in vaccine acceptance). We analyzed the survey responses through three different regression models (see Materials and methods and S3–S14 Tables in S1 File). Table 1 reports, for each model, the average marginal effects (M).

**Table 1:** Summary of regression analysis.

Predictors	Avg. Believability (Model 1)	Vacc. Acceptance (Model 2)	Vacc. Acceptance Grouped (Model 3)
**Control Variables**			
*Perceived Threat*	0.114***(0.095 − 0.133)	0.166***(0.155 − 0.177)	0.147***(0.135 − 0.158)
*Past Vacc.*	–	−0.029**(−0.046 − −0.012)	0.003(−0.014 − 0.020)
*Past Non-Mandatory Vacc.*	–	0.184***(0.170 − 0.199)	0.171***(0.157 − 0.185)
**Independent Variables**			
*Exposure*	0.098***(0.093 − 0.103)	−0.004*(−0.007 − −0.001)	–
*Believability*	–	−0.128***(−0.137 − −0.119)	–
Fact-Checks	−0.010**(−0.015 − −0.004)	0.016***(0.012 − 0.019)	–
**5G Claims**			
*Exposure*	–	–	−0.025**(−0.043 − −0.007)
*Believability*	–	–	−0.002(−0.009 − 0.005)
*Fact-Checks*	–	–	0.007(−0.02 − 0.035)
**DIY Claims**			
*Exposure*	–	–	0.024***(0.017 − 0.031)
*Believability*	–	–	0.001(−0.010 − 0.012)
*Fact-Checks*	–	–	0.005(−0.004 − 0.014)
**Hot&Co Claims**			
*Exposure*	–	–	0.010*(0.001 − 0.019)
*Believability*	–	–	0.012*(0.003 − 0.022)
*Fact-Checks*	–	–	0.009(−0.001 − 0.020)
**Vaccination Claims**			
*Exposure*	–	–	−0.035***(−0.042 − −0.027)
*Believability*	–	–	−0.120***(−0.128 − −0.113)
*Fact-Checks*	–	–	0.032***(0.018 − 0.045)

The table presents the average marginal effects of all main predictors across the three models proposed by the study and their corresponding 95% confidence intervals. The scale of the variables can be referred to in S2 Table in S1 File. Significance marked as *p <.05, **p <.01, ***p <.001. In addition, we present fixed-, mixed-effects, lasso and elastic net model regression coefficients in S3–S14 Tables in S1 File.

For Model 1, we find that exposure to misinformation is positively correlated with overall claim believability (M = 0.098, 95% CI 0.093 − 0.103). A higher perceived threat concerning the pandemic is also associated with higher believability (M = 0.114, 95% CI 0.095 − 0.133). Our findings indicate a weak effect of exposure to fact-checks in believability (M = –0.010, 95% CI -0.015 − -0.004).

The Model 2 results, in which claims are not grouped, show that mere exposure to misinformation is not strongly associated with vaccination willingness (M = –0.004, 95% CI -0.007 − -0.001). However, the perceived believability of false information is associated with vaccine refusal (M = -0.128, 95% CI -0.137 − -0.119). Although statistically significant, the marginal effect size of past-vaccination history is negligibly small, while those who had received a non-mandatory vaccine in the past report higher vaccine acceptance (M = 0.184, 95% 0.170 − 0.199); we observe the same association for those that perceive the pandemic as more threatening (M = 0.166, 95% CI 0.155 − 0.177).

Our final logistic regression model (Model 3), which groups related claims, indicates that increased exposure to vaccine-related misinformation is directly associated with an increased level of vaccination hesitancy (M = -0.035, 95% CI -0.042 − 0.027). A more substantial association was found for the reported believability of vaccination-related claims (M = -0.120, 95% CI -0.128 − -0.113). Our results also show that increased exposure to fact-checked vaccination-related information is correlated with increased vaccine acceptance (M = 0.032, 95% CI 0.018 − 0.045).

**Fig 6:**
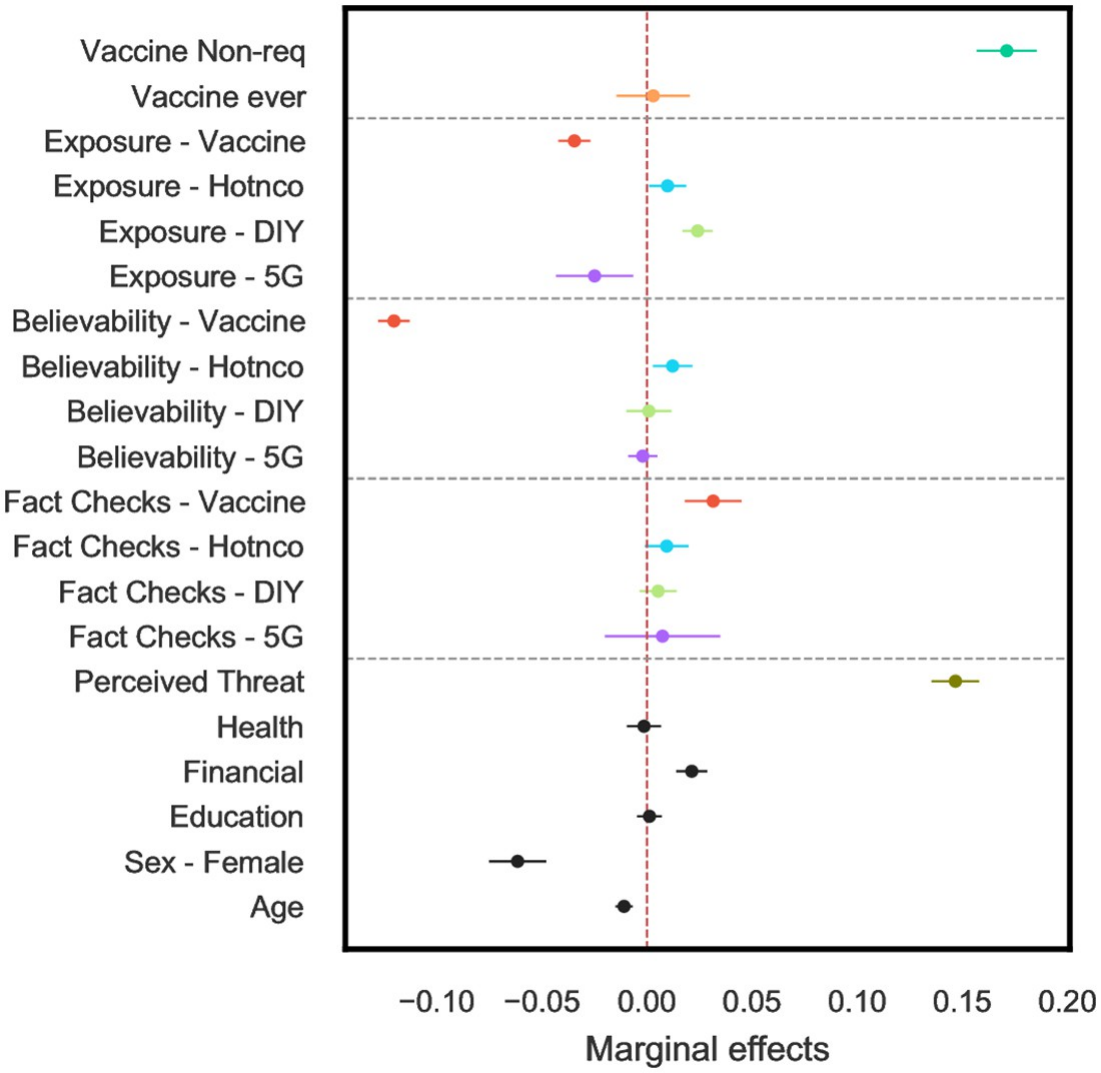
Model 3’s marginal effects of all predictors and their 95% confidence intervals. Variables are color-coded as per the groups. The horizontal dashed lines indicate *Exposure*, *Believability* and *Fact Checks* for different groups.

Aside from the vaccination-related claims, our results suggest that exposure, believability, and fact-checking of other types of misinformation are not strongly associated with vaccine acceptance (see Table 1). Additionally, following our Model 2 results, respondents who feel more threatened by the pandemic are more likely to get vaccinated (M = 0.147, 95% CI 0.135 − 0.158). People with previous experience with non-mandatory vaccines also report higher vaccine acceptance (M = 0.171, 95% CI 0.157 − 0.185), whereas general past vaccination has no statistically significant effect. The relative marginal effects for Model 3 can be visualized in Fig 6.

## Discussion

### Using social media as a survey platform

The present study used the Facebook Advertisement Platform as a recruitment platform for our survey and obtained responses from over 18,000 respondents worldwide. We described our method for collecting demographically diverse survey responses via targeted advertisements for different locations and varying age groups. We also employed a post-stratification weighting scheme (i.e., raking) to correct survey results for non-response and non-coverage. The broad and deep reach of social media and the weighting technique helped us better estimate the infodemic’s worldwide reach compared to other commonly used crowdsourcing platforms.

The Facebook Advertisement Platform is a financially viable choice for conducting global-scale surveys. Our survey, reaching over 50,000 people with 18,407 complete responses, cost US$8,550. Assuming an identical setting (e.g., a median of 11 minutes to complete the survey), a survey of the same scale could have cost over five times, e.g., US$43,741 on Prolific [25] or US$45,870 on Amazon Mechanical Turk [32]. Moreover, the demographic reach would be significantly lower with participants mainly from selected regions. For instance, Amazon Mechanical Turk’s user base predominantly comprises the US and Indian residents [33], and Prolific’s workers reside in OECD countries. We believe the economic viability and broad geographic reach make social media advertising platforms a feasible survey tool for researchers and practitioners.

### Treatment of local versus global rumors

The top-2 rumors, i.e., Drugs and Weather, reached nearly three-fourths of all respondents globally. Besides an inherent appeal of these claims, they might have spread more widely for political reasons. Public figures worldwide downplayed the virus’ impact by stating that it would disappear as temperatures started rising [34]. The potential use of existing drugs like hydroxychloroquine, a malaria drug that has not shown any promising result [35], has been openly promoted as a potential therapy by politicians [36, 37]. These findings exemplify the influence that local public figures can exert on the general public’s information consumption, as shown in a prior study on the public narrative of Ibuprofen’s possible side-effects on coronavirus patients [38].

Another finding of this paper was the uneven spread of rumors across geographic regions (see Fig 3). One of the localized rumors was that saltwater gargling prevents the coronavirus infection (Gargling). This claim had exceptionally high exposure in the Middle East; nearly 90% of respondents from Saudi Arabia and Iraq reported to have seen the claim compared to only around 20% of respondents in Sweden and Finland. Although the rumor’s premise may be harmless, this claim led to tens of infection in South Korea as some churchgoers continued to congregate after spraying saltwater in each other’s mouths [7]. There have been numerous cases where seemingly harmless misinformation swayed people away from official guidelines (e.g., social distancing, washing hands with soap). In addition to tackling globally popular misinformation, local governments could work together with platforms to further debunk claims with a strong regional foothold.

### Algorithmic prioritization of fact-checks

When it comes to prioritizing claims to debunk first or deciding which fact-checks to disseminate widely, fact-checking organizations need to consider both the exposure and the believability of claims. A good example to discuss is 5G, which was seen by 60% of all respondents, yet had low believability. This may indicate that relative to its wide dissemination, its potential harms may not be extensive given that people are not susceptible to believing it.

Based on rumor exposure, fact-check exposure, and believability, we propose a heuristic algorithmic prioritization method to decide which fact-checks to prioritize. We propose an estimate of *blind belief* in a rumor as

$$\begin{array}{c} \left(\text{Rumor}\;\text{Exposure}-\text{Fact}-\text{Check}\;\text{Exposure})\times (\text{Believability}\right) \end{array}$$ (3)

Using the dichotomized value of believability, we can roughly estimate a proxy for how many people may believe a specific claim. The same idea can be used in deciding which rumors to debunk first.

**Fig 7:**
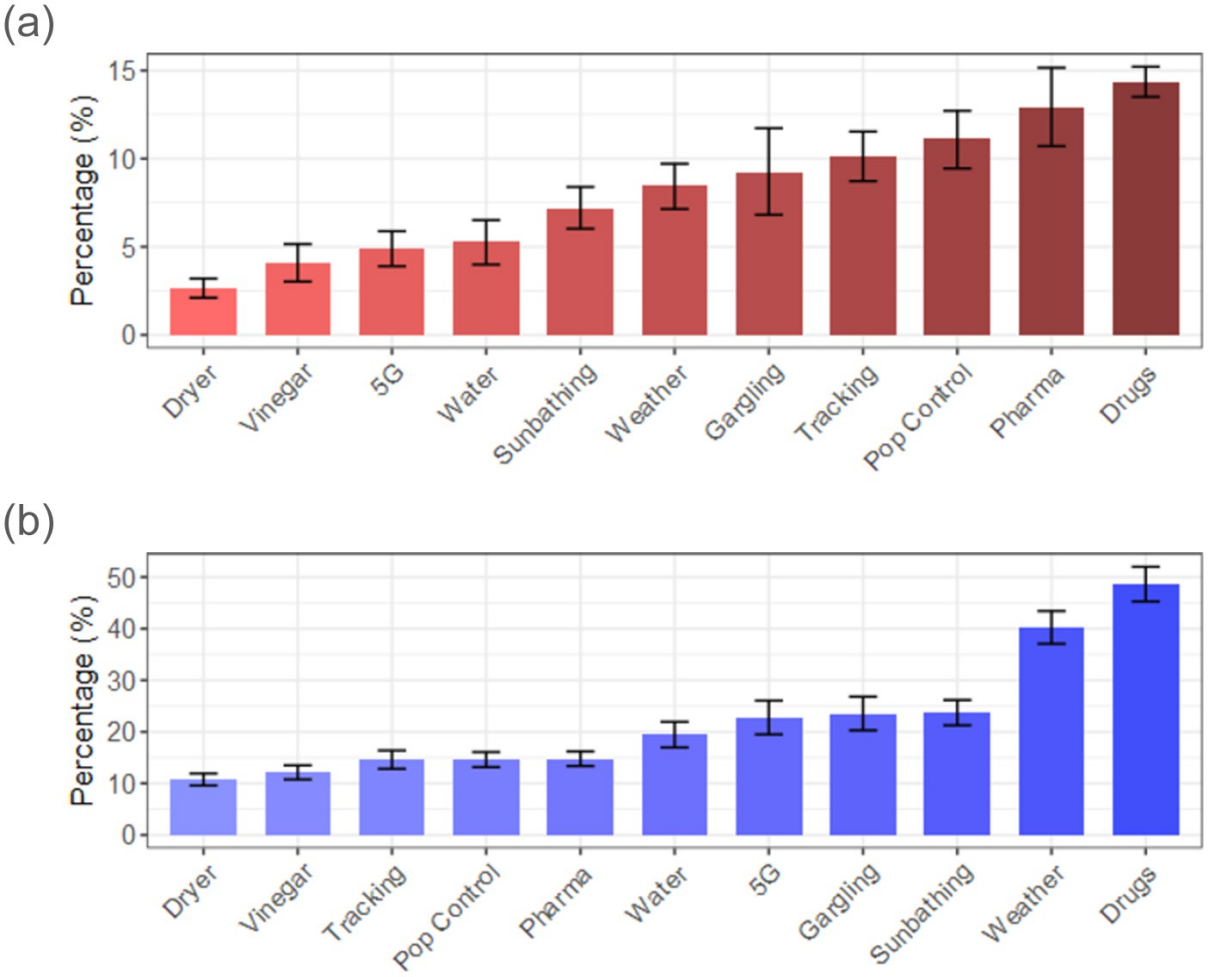
Comparison of claims ranked based on (a) heuristic algorithmic prioritization and (b) how currently practiced. (a) Blind belief scale (% of respondents who will likely believe in claims upon exposure, without having access to fact-checks). (b) Aggregate dissemination percentage of fact-checks.

Fig 7(a) shows the estimated percentage of respondents that might believe each claim after encountering it online without having seen a fact-check (i.e., our proposed heuristic). Drugs exhibits the highest value, followed by the three vaccination-related claims. Although Weather was the second most seen claim, it is ranked sixth in the blind belief scale. Rumors addressing DIY measures against the disease (e.g., Gargling, Sunbath) suggests that less than 10% of the population might believe them without access to fact-checks. 5G is ranked ninth, which is low if compared to its disproportionate large exposure.

In contrast, Fig 7(b) shows the popularity of fact-checks by the weighted percentage of survey participants who have seen them. Note that Drugs ranks at the top as in the algorithmic suggestion. However, our results suggest that the three vaccination-related fact-checks, which the proposed algorithm recommends to be highly prioritized, have not been widely popular in real campaigns. We also see relatively high dissemination for Weather and Sunbath compared to their blind belief potential.

The observation above implies that fact-checking organizations and social media platforms could use simple online tools to elicit users’ perceived believability and identify which claims are more likely to be widely believed. A possibility would be social media platforms presenting prompts to users inquiring whether they find claims believable as soon as they are identified in online networks (e.g., by machine learning methods or reporting functions). Our proposed method builds upon existing automated and human-in-the-loop approaches to detecting misinformation and uses data obtained by these methodologies to prioritize claims that are more likely to be believed by the general public.

We highlight that efforts by fact-checking companies and social media platforms should prioritize those claims that are widely shared *and* perceived to be believable. This prioritization could prove to be incredibly helpful, considering the limited manual fact-checking resources and the relative inaccuracy of automated fact-checking models.

### Underdeveloped countries are more susceptible to the infodemic

Another important finding of this study is that economically disadvantaged countries are exposed more to the infodemic than richer countries. Moreover, respondents from nations with lower GDP per capita generally are more susceptible to believing in misinformation upon exposure. This finding is most prominent for claims that propose DIY preventive measures, such as Gargling and Water. This can be linked to the literature that has found that those more economically disadvantaged are more likely to have limited health literacy [39, 40]. Users from these countries have limited access to healthcare [41, 42], which might make them more receptive to non-conventional health behaviors.

Underdeveloped countries seem to be in higher distress during the pandemic [43], mainly due to a lack of healthcare infrastructure and limited number of health professionals [44]. The spread of the infodemic could increase the burden caused by COVID-19 in these countries, as health information inequalities are known to widen global health disparities [45].

Previous research has indicated a positive correlation between GDP per capita and better living standards, healthcare facilities, and literacy rates; this association is reversed with respect to the unemployment rate and economic hardships [46, 47]. Our findings highlight that the fragile healthcare systems and vulnerable economies of underdeveloped countries not only have to bear the burden of the pandemic but also that of a pronounced infodemic.

A Pew Research Center study has found that social media use is continuously rising in underdeveloped and developing countries [48]. Although people might be exposed to more misinformation as they go online, this also creates opportunities for the dissemination of accurate and debunking information online. Hence, our findings underline the importance of fact-checking in propagating correct information *before* rumors spread in vulnerable countries. Preparing reactive fact-checks alone might not be enough if their spreading potential is smaller than that of rumors and if falsehoods are considered believable. Social media platforms could be used to facilitate the dissemination of such preemptive fact-checks.

### Vaccination-related rumors are popular worldwide

Vaccination-related claims showed no significant difference in exposure between developed and underdeveloped countries. More worryingly, our results suggest that these claims were widely shared online, reporting up to 60% exposure across the countries covered by our study. Our proposed prioritization method also presents these claims as prospects for high belief among the world population. This is a concern for global health as research has found that information delivered through social media can cause COVID-19 vaccine hesitancy [49].

Given the importance of vaccination in the control of the pandemic [12] and the strong influence that anti-vaccination movements exert in online communities [50], we highlight the importance of fighting this type of misinformation, particularly now that coronavirus vaccines are being rolled out. Some online platforms have taken proactive stances on this topic and banned misinformation about COVID-19 vaccines [51, 52]. Our findings highlight the importance of these efforts, and we urge other social platforms to adopt a similar stance on the topic.

### Infodemic and vaccine hesitancy

Our analysis reveals that exposure to misinformation is associated with vaccination decisions. However, we observe that false information’s perceived believability is a much more decisive factor in vaccine acceptance than mere exposure. Susceptibility to believing in misinformation, and consequent belief in an unconfirmed piece of information, could have critical implications on public health behaviors. On the other hand, increased exposure to fact-checked information was associated with a more positive attitude towards the coronavirus vaccine. Although the adverse impact of perceived believability of misinformation in vaccine acceptance is more pronounced than that of fact-checked information, its positive effect highlights the importance of concerted efforts for disseminating accurate and debunking information to the public.

As the claims addressed in this study cover various aspects of the infodemic, we also studied whether different misinformation categories had varying effects on people’s vaccination tendencies. Although increased exposure to vaccination-related false information and associated believability was negatively associated with vaccine acceptance, our results suggest marginally adverse or even positive effects for other types of misinformation (see Fig 6).

These conflicting and marginal results indicate that misinformation not directly addressing vaccination might not be associated with vaccine refusal. For instance, those exposed to more DIY-related claims show higher rates of vaccine acceptance; people adhering to various behavioral measures for their safety might also feel more threatened about the coronavirus and thus may be more willing to accept a vaccine. Another hypothesis is that people interested in personal health and well-being, i.e., arguably more likely to have seen DIY rumors, are active followers of coronavirus-related information to protect themselves from infection. The opposite effect was observed for the 5G rumor; people who have seen this conspiracy theory might also have been exposed to vaccination-related conspiracies [53] and hence show higher vaccine refusal rates.

## Conclusion

To understand how the COVID-19 infodemic has affected different countries worldwide, we conducted a large-scale survey to quantify the public exposure to a wide range of coronavirus-related misinformation and fact-checks. Additionally, we assessed the extent to which people’s susceptibility to misinformation is negatively associated with their acceptance of the coronavirus vaccine. All forty countries examined showed extensively higher exposure to rumors than to their respective fact-checks. Most importantly, our findings indicate that the infodemic could disproportionately hit economically disadvantaged countries the hardest. These vulnerable countries have higher rates of exposure to coronavirus-related rumors and their residents find claims more believable than those of economically developed nations.

Our study indicates that misinformation, particularly to what extent people are open to believing in it, has a negative association with their acceptance of the COVID-19 vaccine. A more fine-grained analysis revealed how vaccination-related claims could contribute to vaccination hesitancy, while other false information does not to correlated with these decisions. Worryingly, our findings indicate that the positive effect of fact-checks on vaccine acceptance is less pronounced than the extent to which the population is susceptible to believing in misinformation.

There are, however, several limitations that might be associated with this work. Although we have designed our study to cover a wide range of coronavirus-related misinformation topics, our list is not comprehensive of the whole infodemic. Future work should address a more extensive list of rumors that were not covered in the current study. We have also conducted our study in a month-long time window. The infodemic is under constant mutation, and future studies should also address the temporal aspect of misinformation. Although we have adopted post-stratification methods to compensate for non-respondents and non-coverage, our results might not be generalizable to those countries with smaller sample sizes. Additionally, our weighting technique, i.e., raking, might be associated with issues like non-convergence under some conditions [54, 55].

We recruited our respondents through social media to cover a wide range of respondents from different world regions and adopted weighting methods to compensate for non-respondents, but our results are not strictly representative of the world population. For instance, we have focused our efforts on economically undeveloped countries (e.g., in Africa), as previous work indicates that developing countries are more vulnerable to communicable diseases [56]. Hence, our respondents do not cover the majority of other countries. Facebook users could also be more susceptible to being exposed to rumors, as false information is rapidly disseminated online. Moreover, we maximized the reach of our survey by translating it into some of the most widely spoken languages, but we did not cover Chinese, Indic, Slavic, and other languages.

Our results in this work are based on indirect measures and self-reported values, which could result in potential confounds and response biases. For instance, mere exposure to debunking information from a news agency might be construed as exposure to misinformation. Other limitations include the social desirability bias [57], where respondents might answer the survey questions in a certain way to be viewed more favorably by others. We note that previous studies have not observed social desirability biases in estimates of compliance with COVID-19 regulations [58]. Hence, our main findings associating rumor exposure and believability with vaccine hesitancy should be consistent with Facebook users’ views.

An influencing factor could also be the terminology used in the survey questionnaire. An example would be the “official source” term used in the question addressing whether they had seen an official source debunking a specific piece of misinformation. Since our survey did not control for the definition of an “official source,” respondents had the liberty to answer according to their understanding, which could have led to some variance and noise in the responses. For future work, these limitations could be mitigated by clearer phrasing. Another design choice was to obtain participants’ perceived believability of COVID-19 claims and not their actual beliefs. In other words, this study measured participants’ susceptibility to believing in misinformation rather than their true belief. Future studies could explore the association between vaccine hesitancy and belief in false claims.

It is also important to consider a possible limitation concerning self-selection biases; participants who chose to take part in the study by clicking on its advertisement might have been particularly interested in the pandemic. Nevertheless, we highlight that previous studies have found no major bias in Facebook samples compared to traditionally administered surveys [59], particularly if correction factors, such as post-stratification weights, are used [30].

The fact that we quantified infodemic spread and its association with vaccine hesitancy at a global scale makes our work truly unique. We present our analysis with individual responses from 40 countries of world, covering the continents of Asia, Africa, Europe, Oceania, and the Americas, with translations in widely spoken local languages. This work is also distinct from existing Anglo-centric research.

We defended a proactive stance in disseminating accurate information and flagging suspicious content before rumors are widely spread and believed. We also highlighted the importance of local efforts in fighting the infodemic as claims might be constrained to specific regions. We discussed how social media platforms could elicit users’ perceived believability of rumors to prioritize which misinformation should be quickly addressed. In addition, we proposed a heuristic prioritization method based on the reach of rumors, fact-checks, and perceived believability to assist decisions of which rumors to prioritize in the fight against the infodemic.

Finally, our findings suggest that accurate information promulgating public awareness about the disease’s risks and side effects is important for widespread vaccine acceptance. We underlined the importance of debunking falsehoods and spreading truths about vaccination given the public susceptibility to and widespread reach of vaccination-related rumors. Additionally, our study indicates that economically vulnerable countries are more susceptible to infodemic. Considering these nations’ more precarious healthcare infrastructure, we defend that public organizations and social media platforms should prioritize underdeveloped and developing countries in their fight against misinformation. Social media is continuously growing in these nations, and platforms should play a key role in suppressing rumors and disseminating facts. We hope findings from this work contribute to public and policy decisions and enable health authorities to better respond to future infodemics.

## Supporting information

S1 File (PDF)
