# Supplementary material for: Misinformation, believability, and vaccine acceptance over 40 countries: Takeaways from the initial phase of the COVID-19 infodemic
Source: PLoS One. 2022 Feb 9;17(2):e0263381. doi: 10.1371/journal.pone.0263381 (PMC8827463; doi:10.1371/journal.pone.0263381)

Misinformation, Believability, and Vaccine Acceptance Over 40 Countries: Takeaways From the Initial Phase of The COVID-19 Infodemic

Karandeep Singh[1¶], Gabriel Lima[2,1¶], Meeyoung Cha[1,2*], Chiyoung Cha[3*], Juhi Kulshrestha[4], Yong-Yeol Ahn[5,6,7], Onur Varol[8]

1 Institute for Basic Science, Daejeon, 34126, South Korea
2 School of Computing, KAIST, Daejeon, 34141, South Korea
3 College of Nursing, Graduate Program in System Health Science and Engineering, Ewha Womans University, Seoul, 03760, South Korea
4 Department of Politics and Public Administration, University of Konstanz, 78457 Konstanz, Germany
5 Center for Complex Networks and Systems Research, Luddy School of Informatics, Computing, and Engineering, Indiana University, IN 47408, USA
6 Indiana University Network Science Institute, Indiana University, IN 47408, USA
7 Connection Science, Massachusetts Institute of Technology, MA 02139, USA
8 Faculty of Engineering and Natural Sciences, Sabanci University, Istanbul 34956, Turkey

¶These authors contributed equally to this work.
* mcha@ibs.re.kr, chiyoung@ewha.ac.kr

Supporting Information

S1 - S14 Tables

Table S1. Demographic distribution of study participants by country.

		Gender	Age						Education		
Country	N	Female	18-24	25-34	35-44	45-54	55-64	65+	HS or Lower	BS or Assoc. Degree	Grad. Degree
AGO	355	54.9%	29%	22.8%	18.9%	11.5%	13%	4.8%	50.7%	33.8%	15.5%
ARG	1086	25.5%	8.1%	7.1%	21%	15.8%	29.9%	18%	48.2%	9.9%	42%
BFA	75	77.3%	16%	21.3%	22.7%	12%	18.7%	9.3%	24%	32%	44%
BHR	31	71%	9.7%	29%	22.6%	12.9%	12.9%	12.9%	16.1%	71%	12.9%
BRA	701	23.4%	29%	11.4%	22.8%	9.7%	16.5%	10.6%	54.1%	30%	16%
CAN	871	36.4%	10.8%	7.9%	13.2%	11.1%	27.2%	29.7%	48.7%	34.8%	16.5%
CHL	569	19%	15.6%	7.4%	26%	17%	21.1%	12.8%	52.4%	8.8%	38.8%
CMR	93	58.1%	10.8%	23.7%	30.1%	11.8%	20.4%	3.2%	25.8%	24.7%	49.5%
DEU	631	41%	31.7%	9.4%	14.4%	17.4%	16.6%	10.5%	43.6%	26.9%	29.5%
DOM	481	24.3%	21.6%	24.5%	23.5%	13.5%	11.2%	5.6%	36.8%	12.5%	50.7%
DZA	1061	66.6%	22.8%	33.3%	29.9%	10.7%	3%	0.3%	19.2%	50.9%	29.9%
EGY	639	60.1%	33.6%	26%	23.6%	10.6%	4.2%	1.9%	30.4%	57%	12.7%
ESP	363	35.8%	16.5%	11.3%	21.8%	16.8%	20.7%	12.9%	32%	24%	44.1%
FIN	418	40%	28.9%	15.6%	19.1%	17.7%	9.3%	9.3%	45.2%	30.6%	24.2%
FRA	728	37.5%	14.7%	11.5%	19.4%	13.7%	19.9%	20.7%	36.4%	19.1%	44.5%
GBR	614	30.6%	14.5%	7.5%	21.8%	6.8%	16.8%	32.6%	47.9%	31.4%	20.7%
GTM	681	37.9%	21.7%	18.6%	23.1%	16.4%	13.1%	7%	46.1%	14.7%	39.2%
IND	229	63.3%	24%	8.3%	11.8%	16.2%	14.8%	24.9%	21%	29.3%	49.8%
IRQ	883	75.9%	22.7%	32.2%	22.2%	16%	4.9%	2.2%	29%	61.7%	9.3%
KEN	207	51.7%	14.5%	31.4%	29.5%	15.5%	6.8%	2.4%	42.5%	38.6%	18.8%
MAR	454	70.7%	28%	32.6%	23.3%	8.1%	5.1%	2.9%	26.9%	46.9%	26.2%
MOZ	414	57.7%	19.1%	31.2%	20.5%	13.5%	12.3%	3.4%	42.5%	46.1%	11.4%
MYS	241	41.9%	32.8%	21.2%	15.8%	10.8%	14.1%	5.4%	44.8%	36.1%	19.1%
NER	60	85%	13.3%	25%	20%	31.7%	10%	0%	23.3%	25%	51.7%
NGA	300	66.7%	16.7%	30.3%	27.7%	14.3%	9.7%	1.3%	21%	52.7%	26.3%
NIC	528	40.2%	25.8%	19.7%	17.6%	14.8%	13.6%	8.5%	34.1%	8.7%	57.2%
PHL	621	51%	24.5%	12.9%	19.6%	11.8%	17.2%	14%	32%	49.9%	18%
PNG	51	78.4%	9.8%	5.9%	31.4%	19.6%	21.6%	11.8%	47.1%	35.3%	17.6%
PRT	520	28.7%	28.7%	9%	26.9%	12.9%	12.1%	10.4%	49.8%	37.7%	12.5%
RWA	106	72.6%	6.6%	21.7%	30.2%	25.5%	12.3%	3.8%	12.3%	60.4%	27.4%
SAU	76	52.6%	6.6%	25%	30.3%	19.7%	14.5%	3.9%	22.4%	53.9%	23.7%
SEN	234	50.4%	10.3%	18.4%	17.1%	14.5%	23.5%	16.2%	28.6%	19.7%	51.7%
SWE	779	40.9%	24.4%	15.5%	22.1%	14.8%	13.1%	10.1%	40.8%	30.7%	28.5%
TTO	353	32%	11.3%	12.2%	24.1%	20.1%	19.3%	13%	39.9%	40.8%	19.3%
TUN	436	54.8%	29.6%	27.1%	25.7%	12.4%	3.4%	1.8%	23.9%	47.5%	28.7%
URY	542	23.1%	11.3%	6.5%	18.8%	18.6%	26.6%	18.3%	47%	19.7%	33.2%
USA	378	40.5%	14.8%	8.2%	12.7%	10.8%	22%	31.5%	37.3%	38.6%	24.1%
VEN	459	35.1%	10%	10.5%	15.3%	18.7%	25.3%	20.3%	27.5%	13.9%	58.6%
YEM	342	68.1%	27.2%	32.7%	25.4%	11.4%	2.6%	0.6%	28.4%	60.5%	11.1%
ZAF	704	21.6%	9.7%	8.9%	18.9%	17.5%	26.3%	18.8%	53.7%	27.1%	19.2%

Table S2. Coding rules used for regression analysis of believability and vaccine acceptance.

Variable	Values	Usage
Age	0 – 5	Coded Values
Sex	0 & 1	Coded Values
Education	0 – 4	Coded Values
Financial	-2 – 2	Coded Values
Vaccine History	0 & 1	Factor
Perceived Threat	0 – 3	Mean
Exposure	0 & 1	Count
Fact-Checks	0 & 1	Count
Believability	-2 – 2	Mean
Vaccine Decision	0 & 1	Factor

Table S3. Model 1 regression results. Average believability is predicted from exposure to false claims and their respective fact-checks. Standard errors are presented between parenthesis. Significance marked as * P<0.1; ** P<0.05; *** P<0.01.

	Dependent Variable:
	Average Believability
Constant	-1.399^{***}
	(0.032)
Sex	-0.034^{***}
	(0.011)
Age	-0.009^{**}
	(0.003)
Education	-0.027^{***}
	(0.005)
Financial Status	-0.111^{***}
	(0.006)
Health Status	0.071^{***}
	(0.007)
Perceived Threat	0.114^{***}
	(0.010)
Fact-Checks	-0.068^{***}
	(0.007)
Exposure	0.075^{***}
	(0.003)
Exposure × Fact-Checks	0.010^{***}
	(0.001)
Observations	18,314
R^2	0.129

Table S4. Model 1 mixed-regression results. Average believability is predicted from exposure to false claims and their respective fact-checks. We include the respondent's residence country as a random effect. Standard errors are presented between parenthesis. Significance marked as * P<0.1; ** P<0.05; *** P<0.01.

	Dependent Variable:
	Average Believability
Constant	-0.774^{***}
	(0.061)
Sex	0.077^{***}
	(0.011)
Age	0.032^{***}
	(0.003)
Education	-0.058^{***}
	(0.005)
Financial Status	-0.091^{***}
	(0.006)
Health Status	0.018^{***}
	(0.007)
Perceived Threat	-0.085^{***}
	(0.010)
Fact-Checks	-0.067^{***}
	(0.007)
Exposure	0.051^{***}
	(0.003)
Exposure × Fact-Checks	0.010^{***}
	(0.001)
Country-Level Random Effects	Yes
Observations	18,314
R^2	0.247

Table S5. Model 1 elastic regression results. Average believability is predicted from exposure to false claims and their respective fact-checks.

	Dependent Variable:
	Average Believability
Constant	−1.096
Sex - Male	0.039
Sex - Female	−0.002
Age	−0.006
Education	−0.014
Financial Status	−0.058
Health Status	0.043
Perceived Threat	0.103
Fact-Checks	−0.042
Exposure	0.049
Exposure × Fact-Checks	0.007
Observations	18,314

Table S6. Model 1 lasso regression results. Average believability is predicted from exposure to false claims and their respective fact-checks.

	Dependent Variable:
	Average Believability
Constant	−1.100
Sex - Male	0.040
Sex - Female	0.000
Age	−0.006
Education	−0.013
Financial Status	−0.058
Health Status	0.042
Perceived Threat	0.102
Fact-Checks	−0.039
Exposure	0.050
Exposure × Fact-Checks	0.006
Observations	18,314

Table S7. Model 2 regression results. Each respondents' vaccine decision is predicted based on exposure to false claims and their fact-checks, believability, perceived threat, and vaccination history. Standard errors are presented between parenthesis. Significance marked as * P<0.1; ** P<0.05; *** P<0.01.

	Dependent Variable:
	Vaccine Acceptance
Constant	-2.067^{***}
	(0.101)
Sex	-0.307^{***}
	(0.033)
Age	-0.051^{***}
	(0.010)
Education	0.019
	(0.014)
Financial Status	0.145^{***}
	(0.018)
Health Status	-0.007
	(0.020)
Past Vaccination	-0.195^{***}
	(0.046)
Past Non-Mandatory Vaccination	0.737^{***}
	(0.073)
Past Vaccination × Past Non-Mandatory Vaccination	0.129
	(0.082)
Perceived Threat	0.775^{***}
	(0.029)
Fact-Checks	0.061^{***}
	(0.021)
Exposure	-0.023^{**}
	(0.009)
Average Believability	-0.598^{***}
	(0.023)
Exposure × Fact-Checks	0.002
	(0.003)
Observations	18,314
R^2	0.132

Table S8. Model 2 mixed-regression results. Each respondents' vaccine decision is predicted based on exposure to false claims and their fact-checks, believability, perceived threat, and vaccination history. We include the respondent's residence country as a random effect. Standard errors are presented between parenthesis. Significance marked as * P<0.1; ** P<0.05; *** P<0.01.

	Dependent Variable:
	Vaccine Acceptance
Constant	-1.859^{***}
	(0.134)
Sex	-0.298^{***}
	(0.035)
Age	-0.025^{**}
	(0.011)
Education	0.008
	(0.015)
Financial Status	0.151^{***}
	(0.019)
Health Status	-0.064^{***}
	(0.021)
Past Vaccination	-0.155^{***}
	(0.052)
Past Non-Mandatory Vaccination	0.611^{***}
	(0.076)
Past Vaccination × Past Non-Mandatory Vaccination	0.273^{***}
	(0.085)
Perceived Threat	0.643^{***}
	(0.032)
Fact-Checks	0.050^{**}
	(0.022)
Exposure	-0.028^{***}
	(0.009)
Average Believability	-0.689^{***}
	(0.025)
Exposure × Fact-Checks	0.003
	(0.003)
Country-Level Random Effects	Yes
Observations	18,314
R^2	0.169

Table S9. Model 2 elastic regression results. Each respondents' vaccine decision is predicted based on exposure to false claims and their fact-checks, believability, perceived threat, and vaccination history.

	Dependent Variable:
	Vaccine Acceptance
Constant	−2.067
Sex - Male	0.148
Sex - Female	−0.133
Age	−0.043
Education	0.015
Financial Status	0.127
Health Status	−0.001
Past Vaccination	−0.173
Past Non-Mandatory Vaccination	0.650
Past Vaccination × Past Non-Mandatory Vaccination	0.181
Perceived Threat	0.712
Fact-Checks	0.056
Exposure	−0.018
Average Believability	−0.555
Exposure × Fact-Checks	0.002
Observations	18,314

Table S10. Model 2 lasso regression results. Each respondents' vaccine decision is predicted based on exposure to false claims and their fact-checks, believability, perceived threat, and vaccination history.

	Dependent Variable:
	Vaccine Acceptance
Constant	−2.370
Sex - Male	0.296
Sex - Female	0.000
Age	−0.047
Education	0.015
Financial Status	0.137
Health Status	0.000
Past Vaccination	−0.174
Past Non-Mandatory Vaccination	0.745
Past Vaccination × Past Non-Mandatory Vaccination	0.103
Perceived Threat	0.761
Fact-Checks	0.068
Exposure	−0.017
Average Believability	−0.591
Exposure × Fact-Checks	0.001
Observations	18,314

Table S11. Model 3 regression results. Each respondents' vaccine decision is predicted based on exposure to false claims and their fact-checks, believability, perceived threat, and vaccination history. Claims are grouped into their respective topic. Standard errors are presented between parenthesis. Significance marked as * P<0.1; ** P<0.05; *** P<0.01.

	Dependent Variable:
	Vaccine Acceptance
Constant	−1.997***
	(0.104)
Sex	−0.304***
	(0.035)
Age	−0.054***
	(0.010)
Education	0.006
	(0.015)
Financial Status	0.105***
	(0.019)
Health Status	−0.007
	(0.020)
Past Vaccination	−0.040
	(0.048)
Past Non-Mandatory Vaccination	0.730***
	(0.075)
Past Vaccination × Past Non-Mandatory Vaccination	0.117
	(0.084)
Perceived Threat	0.724***
	(0.030)
Exposure to Vaccination-Related Claims	−0.164***
	(0.019)
Fact-Checks of Vaccination-Related Claims	0.182**
	(0.073)
Average Believability of Vaccination-Related Claims	−0.594***
	(0.021)
Exposure × Fact-Checks - Vaccination-Related Claims	−0.016
	(0.028)
Exposure to DIY Claims	0.105***
	(0.021)
Fact-Checks of DIY Claims	−0.004
	(0.045)
Average Believability of DIY Claims	0.005
	(0.027)
Exposure × Fact-Checks - DIY Claims	0.014
	(0.015)
Exposure to Hot&Co Claims	0.054**
	(0.025)
Fact-Checks of Hot&Co Claims	0.058
	(0.050)
Average Believability of Hot&Co Claims	0.060**
	(0.024)
Exposure × Fact-Checks - Hot&Co Claims	−0.007
	(0.023)
Exposure to 5G Claim	−0.131***
	(0.040)
Fact-Check of 5G Claim	0.014
	(0.148)
Average Believability of 5G Claim	−0.010
	(0.017)
Exposure × Fact-Checks - 5G Claim	0.038
	(0.154)
Observations	18,314
R^2	0.178

Table S12. Model 3 mixed-regression results. Each respondents' vaccine decision is predicted based on exposure to false claims and their fact-checks, believability, perceived threat, and vaccination history. Claims are grouped into their respective topic. We include the respondent's residence country as a random effect. Standard errors are presented between parenthesis. Significance marked as * P<0.1; ** P<0.05; *** P<0.01.

	Dependent Variable:
	Vaccine Acceptance
Constant	−1.777***
	(0.134)
Sex	−0.320***
	(0.037)
Age	−0.042***
	(0.011)
Education	0.001
	(0.016)
Financial Status	0.118***
	(0.020)
Health Status	−0.062***
	(0.021)
Past Vaccination	−0.110**
	(0.053)
Past Non-Mandatory Vaccination	0.625***
	(0.078)
Past Vaccination × Past Non-Mandatory Vaccination	0.213**
	(0.087)
Perceived Threat	0.626***
	(0.033)
Exposure to Vaccination-Related Claims	−0.164***
	(0.020)
Fact-Checks of Vaccination-Related Claims	0.175**
	(0.074)
Average Believability of	−0.616***
	(0.021)
Exposure × Fact-Checks - Vaccination-Related Claims	−0.011
	(0.028)
Exposure to DIY Claims	0.065***
	(0.022)
Fact-Checks of DIY Claims	0.015
	(0.046)
Average Believability of DIY Claims	−0.025
	(0.028)
Exposure × Fact-Checks - DIY Claims	0.012
	(0.015)
Exposure to Hot&Co Claims	0.084***
	(0.026)
Fact-Checks of Hot&Co Claims	0.027
	(0.051)
Average Believability of Hot&Co Claims	0.070***
	(0.024)
Exposure × Fact-Checks - Hot&Co Claims	−0.005
	(0.023)
Exposure to 5G Claim	−0.090**
	(0.042)
Fact-Checks of 5G Claim	0.036
	(0.152)
Average Believability of 5G Claim	−0.043**
	(0.018)
Exposure × Fact-Checks - 5G Claim	0.061
	(0.157)
Country-Level Random Effects	Yes
Observations	18,314
R^2	0.208

Table S13. Model 3 elastic regression results. Each respondents' vaccine decision is predicted based on exposure to false claims and their fact-checks, believability, perceived threat, and vaccination history. Claims are grouped into their respective topic.

	Dependent Variable:
	Vaccine Acceptance
Constant	−2.210
Sex - Male	0.248
Sex - Female	−0.045
Age	−0.050
Education	0.002
Financial Status	0.097
Health Status	—
Past Vaccination	−0.025
Past Non-Mandatory Vaccination	0.730
Past Vaccination × Past Non-Mandatory Vaccination	0.094
Perceived Threat	0.709
Exposure to Vaccination-Related Claims	−0.161
Fact-Checks of Vaccination-Related Claims	0.133
Average Believability of	−0.582
Exposure × Fact-Checks - Vaccination-Related Claims	—
Exposure to DIY Claims	0.103
Fact-Checks of DIY Claims	0.002
Average Believability of DIY Claims	—
Exposure × Fact-Checks - DIY Claims	0.012
Exposure to Hot&Co Claims	0.046
Fact-Checks of Hot&Co Claims	0.043
Average Believability of Hot&Co Claims	0.052
Exposure × Fact-Checks - Hot&Co Claims	—
Exposure to 5G Claim	−0.116
Fact-Checks of 5G Claim	0.012
Average Believability of 5G Claim	−0.008
Exposure × Fact-Checks - 5G Claim	0.024
Observations	18,314

Table S14. Model 3 lasso regression results. Each respondents' vaccine decision is predicted based on exposure to false claims and their fact-checks, believability, perceived threat, and vaccination history. Claims are grouped into their respective topic.

	Dependent Variable:
	Vaccine Acceptance
Constant	−2.267
Sex - Male	0.294
Sex - Female	0.000
Age	−0.051
Education	0.002
Financial Status	0.098
Health Status	—
Past Vaccination	−0.024
Past Non-Mandatory Vaccination	0.739
Past Vaccination × Past Non-Mandatory Vaccination	0.087
Perceived Threat	0.713
Exposure to Vaccination-Related Claims	−0.162
Fact-Checks of Vaccination-Related Claims	0.134
Average Believability of	−0.586
Exposure × Fact-Checks - Vaccination-Related Claims	—
Exposure to DIY Claims	0.103
Fact-Checks of DIY Claims	0.000
Average Believability of DIY Claims	—
Exposure × Fact-Checks - DIY Claims	0.013
Exposure to Hot&Co Claims	0.046
Fact-Checks of Hot&Co Claims	0.043
Average Believability of Hot&Co Claims	0.054
Exposure × Fact-Checks - Hot&Co Claims	—
Exposure to 5G Claim	−0.117
Fact-Checks of 5G Claim	0.030
Average Believability of 5G Claim	−0.007
Exposure × Fact-Checks - 5G Claim	0.007
Observations	18,314

Fig S1. Country-level exposure to misinformation (pink) and fact-checks (purple)
The plot indicates the percentage of participants who have seen each claim and its corresponding fact-check in a specific country covered by our study. The numbers are calculated after post-stratification weighting by the process of raking. The radial axis represents the percentages ranging from 0 to 100. In the angular axis, distinct claims representing similar notions, i.e., vaccination-related claims, are arranged together. The titles of the plots are each country's ISO 3166-1 alpha-3 codes.

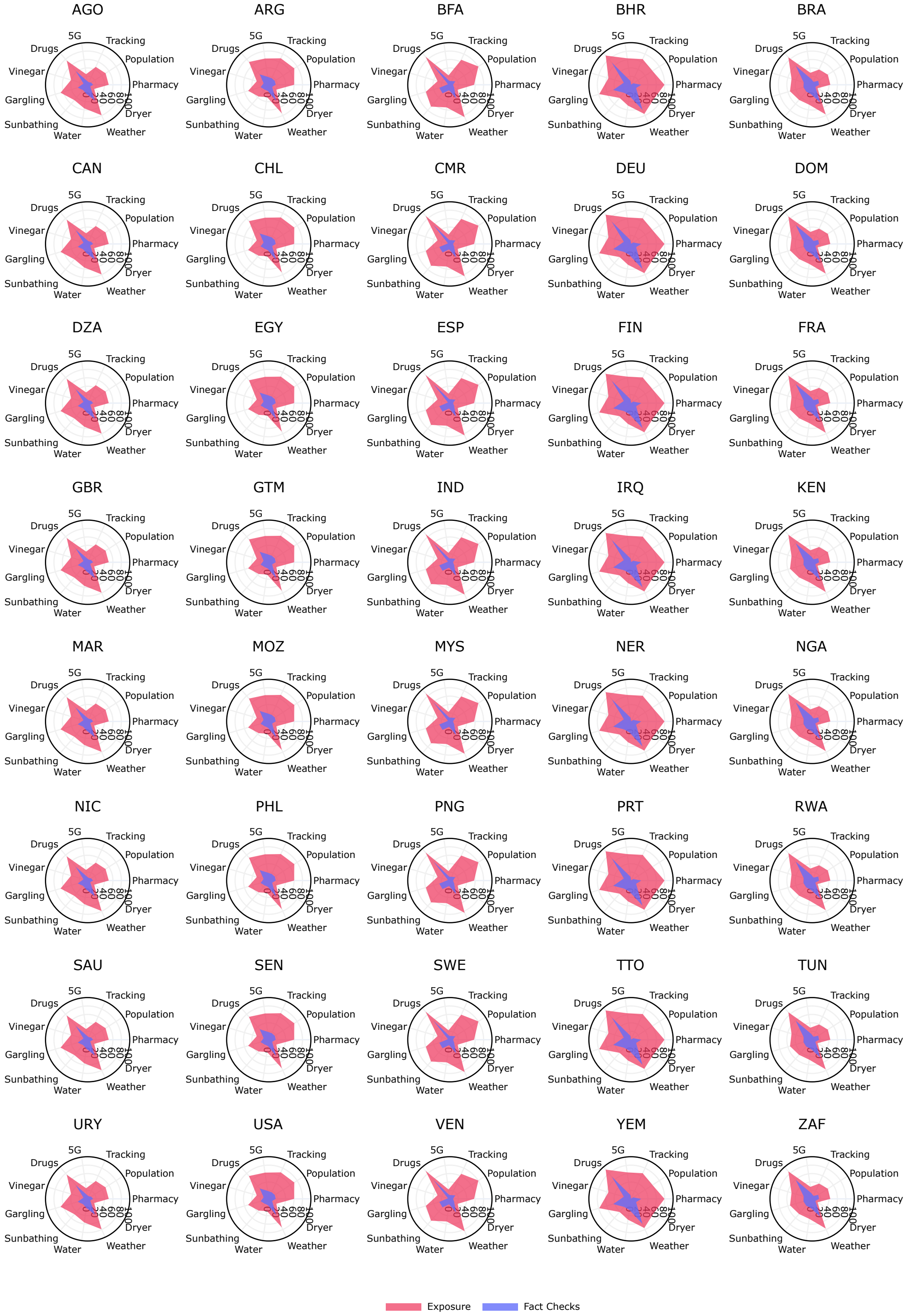

Supplement: S1 File — (PDF) [file pone.0263381.s001.pdf]
